# Supplementary material for: Metabolic Capacity of the Antarctic Cyanobacterium Phormidium pseudopriestleyi That Sustains Oxygenic Photosynthesis in the Presence of Hydrogen Sulfide
Source: Genes (Basel). 2021 Mar 16;12(3):426. doi: 10.3390/genes12030426 (PMC8002359; doi:10.3390/genes12030426)
Supplement: Supplementary file 1 [file genes-12-00426-s001.pdf]

PHOTOSYNTHESIS

Photosystem II

PsbA	yes	Photosystem II P680 reaction center D1 protein
PsbD	yes	Photosystem II P680 reaction center D2 protein
PsbC	yes	Photosystem II CP43 chlorophyll apoprotein
PsbB	yes	Photosystem II CP47 chlorophyll apoprotein
PsbE	yes	Photosystem II cytochrome b559 subunit alpha
PsbF	yes	Photosystem II cytochrome b559 subunit beta
PsbL	yes	Photosystem II PsbL protein
PsbJ	yes	Photosystem II PsbJ protein
PsbK	yes	Photosystem II PsbK protein
PsbM	yes	Photosystem II PsbM protein
PsbH	yes	Photosystem II PsbH protein
PsbI	yes	Photosystem II PsbI protein
PsbO	yes	Photosystem II oxygen-evolving enhancer protein 1
PsbP	yes	Photosystem II oxygen-evolving enhancer protein 2
PsbQ	no	Photosystem II oxygen-evolving enhancer protein 3
PsbR	no	Photosystem II 10 kDa protein
PsbS	no	Photosystem II 22kDa protein
PsbT	yes	Photosystem II PsbT protein
PsbU	yes	Photosystem II PsbU protein
PsbV	yes	Photosystem II cytochrome c550
PsbW	no	Photosystem II PsbW protein
PsbX	yes	Photosystem II PsbX protein
PsbY	yes	Photosystem II PsbY protein
PsbZ	yes	Photosystem II PsbZ protein
Psb27	yes	Photosystem II Psb27 protein
Psb28	yes	Photosystem II 13kDa protein
Psb28-2	no	Photosystem II Psb28-2 protein

Photosystem I

PsaA	yes	Photosystem I P700 chlorophyll a apoprotein A1
PsaB	yes	Photosystem I P700 chlorophyll a apoprotein A2
PsaC	yes	Photosystem I subunit VII
PsaD	yes	Photosystem I subunit II
PsaE	yes	Photosystem I subunit IV
PsaF	yes	Photosystem I subunit III
PsaG	no	Photosystem I subunit V
PsaH	no	Photosystem I subunit VI
PsaI	yes	Photosystem I subunit VIII
PsaJ	no	Photosystem I subunit IX

PsaK	yes	Photosystem I subunit X
PsaL	yes	Photosystem I subunit XI
PsaM	yes	Photosystem I subunit XII
PsaN	no	Photosstem I subunit PsaN
PsaO	no	Photosystem I ubunit PsaO
PsaX	no	Photosystem I 4.8kDa protein
Cytochrome b6/f complex		
PetB	yes	cytochrome b6
PetD	yes	cytohrome b6-f complex subunit 4
PetA	yes	apocytochrome f
PetC	yes	cytochrome b6-f complex iron-sulfur subunit
PetL	no	cytochrome b6-f complex subunit 6
PetM	no	cytochrome b6-f subunit 7
PetN	no	cytochrome b6-f complex subunit 8
PetG	no	cytochrome b6-f complex subunit 5
Photosynthetic electron transport		
PetE	no	plastocyanin
PetF	yes	ferredoxin
PetH	yes	ferredoxin-NaDP+ reductase
PetJ	yes	cytochrome c6
F-type ATPase		
atpD	yes	H+/Na+ transporting ATPase subunit beta
atpA	yes	F-type H+/Na+ transporting ATPase subunit alpha
atpG	yes	H+ transporting ATPase subunit gamma
atpH	yes	F-type H+ transporting ATPase subunit delta
atpC	yes	F-type H+ transporting ATPase subunit epsilon
atpE	yes	F-type H+ transportig ATPase subunit c
atpB	yes	F-type H+ transporting ATPase subunit a
atpF	yes	F-tyoe H+ transporting ATPase subunit b
